# Post-operative pain and associated factors after cesarean section at Hawassa University Comprehensive Specialized Hospital, Hawassa, Ethiopia: A cross-sectional study

**DOI:** 10.1016/j.amsu.2022.104321

**Published:** 2022-08-09

**Authors:** Ibrahim Hussen, Misganaw Worku, Dereje Geleta, Abbas Ahmed Mahamed, Mesfin Abebe, Wondwosen Molla, Aregahegn Wudneh, Tasfaye Temesgen, Zerihun Figa, Muhiddin Tadesse

**Affiliations:** a Department of Obstetrics and Gynecology, College of Medicine and Health Sciences, Dilla University, Dilla, Ethiopia; b Department of Obstetrics and Gynecology, College of Medicine and Health Sciences, Hawassa University, Hawassa, Ethiopia; c School of Public Health, College of Medicine and Health Sciences, Hawassa University, Hawassa, Ethiopia; d Department of Midwifery, College of Medicine and Health Sciences, Dilla University, Dilla, Ethiopia; e Department of Anesthesiology, College of Medicine and Health Sciences, Dilla University, Dilla, Ethiopia

**Keywords:** Post-operative pain, Associated factors, Cesarean section, Ethiopia

## Abstract

**Background:**

The most frequent obstetric surgery both in Ethiopia and around the world is the cesarean section (CS). Postoperative pain that is not well managed can have a major negative impact on surgical patient morbidity, delaying healing and the return to normal daily activities. Even though the cesarean section is one of the most commonly performed operations, postoperative pain after cesarean section and associated factors has not been studied.

**Objective:**

To assess the magnitude and factors associated with postoperative pain after cesarean section at Hawassa University Comprehensive Specialized Hospital, Hawassa, Ethiopia.

**Methods:**

From February 1 to September 30 in 2021, a hospital-based cross-sectional study was undertaken among women who underwent cesarean deliveries at Hawassa University Comprehensive Specialized Hospital. The patient's medical file was read, and information was gathered from them using a structured questionnaire and checklist. The information was prepared for analysis by being cleaned, coded, and put into EPI Data version 3.1 before being exported to SPSS version 20. The prevalence rate and socio-demographic details were displayed using descriptive statistics. Bivariate and multivariable logistic regression analysis was done to identify the associated factors. Variables with a p-value of <0.05 were considered statistically significant.

**Results:**

The magnitude of moderate to severe post-operative pain after a cesarean section was 89.8% (95% CI 84.7, 93.5). Duration of procedure (AOR: 3.62, 95% CI: 1.33, 15.85), type of anesthesia (AOR: 2.38, 95% CI: 1.31, 8.71), and type of analgesics administered (AOR: 2.3, 95% CI: 1.28, 19.21) were significantly associated with moderate to severe post-operative pain.

**Conclusion:**

In this study a significant number of parturient in this study reported moderate to severe post-cesarean pain within 24 h. The duration of the procedure, the type of anesthesia used, and the type of analgesics administered were all found to be significantly associated with postoperative pain after cesarean section.

## Highlights

• The magnitude of post-operative pain after a caesarean section was 89.8%.
• About 84.2% reported moderate to severe pain at 24 h after surgery.
• Duration of procedure was significantly associated to postoperative pain.
• Anesthesia type used was found to be significantly associated to postoperative pain.

## 1 Background

The most frequent issue following surgery is discomfort. Pain is a sensory and emotional experience that is influenced by physiological, sensory, affective, cognitive, sociocultural, and behavioral aspects [1]. Although pain is an inevitable component of the healing process after surgery, it is frequently not managed properly, which can have negative effects. Untreated postoperative pain can result in clinical and psychological changes that impair quality of life while raising morbidity and death [2].

Inadequately treated postoperative pain can significantly contribute to surgical patient morbidity, resulting in a delay in inpatient recovery and the ability to return to daily functional activities [3]. Studies over the last three to four decades have repeatedly confirmed that 20–80% undergoing surgery suffer from inadequately treated pain [4,5], and pain is classified as a serious public health problem both in the developed [6] and in developing countries [[7], [8], [9]].

In Africa, the issue of pain has largely been studied in relation to HIV/AIDS and cancer [[10], [11], [12]], even though the pain from surgical procedures is a far greater burden. According to a Human Rights Watch report, only 10% of these patients can receive optimal pain management [13]. Previous research found that parturient who had CS had a 78.4–92% chance of experiencing moderate to severe pain. However, very little is known about the magnitude of post-cesarean section pain and the factors that contribute to it in developing countries, particularly in Sub-Saharan Africa [14].

According to studies conducted in Jimma and Addis Ababa, the incidence of postoperative pain was 88.2% among surgically treated patients in Ethiopia [15]. The incidence of moderate to severe postoperative pain after a cesarean section was found to be 85.5% in a study conducted at the University of Gondar in Northwest Ethiopia (13). A distinguishing feature of a cesarean section in comparison to other major laparotomies is not only the eagerness but also the requirement for a rapid and safe interaction between patients and their infants shortly after delivery [16]. Inadequately controlled pain in the postoperative period can lead to the development of chronic pain [17]. Unrelieved pain can result in negative consequences affecting patients' psychological and physiological functions [18], interrupting wound healing and delaying patient discharge [19] with subsequent impaired quality of an individual's life [20]. In Ethiopia and around the world, the cesarean section (CS) is the most common obstetric procedure. Despite significant advances in perioperative medicine, a significant proportion of patients continue to experience severe pain following major surgery. Postoperative pain is associated with serious negative outcomes that are costly to both patients and society [21]. Even though the caesarean section is one of the most commonly performed operations, postoperative pain after caesarean section and associated factors has not been well studied. The aims of this study was assess the magnitude and associated factors of postoperative pain after cesarean delivery at Hawassa comprehensive specialized hospital, Hawassa, Ethiopia, 2021.

## 2 Methods and materials

### 2.1 Study area, design, and period

An institutional-based cross-sectional study was conducted at Hawassa University comprehensive specialized Hospital from February 1, 2021, to September 30, 2021. Hawassa is a town in Ethiopia's Sidama region, approximately 273 km from Addis Ababa, the country's capital. The city has seven hospitals, five of which are private and two of which are public. HUCSH is one of the federal ministries of health's tertiary referral hospitals, as well as a teaching hospital for Hawassa University's College of Medicine and Health Sciences. In its catchment area, the hospital serves 18 million people. It has a total of 400 beds and receives approximately 5000 outpatient and emergency visits per month.

### 2.2 Population

All women who gave birth through cesarean delivery at HUCSH were the source population whereas randomly selected women who gave birth by cesarean-section and were available during the data collection period were the study population. Women who were alert and oriented, willing to participate in the study, exposed to general or spinal anesthesia, are on their first post postoperative in the study period at HUCSH were included in the study. Those mothers who were in critically ill conditions, and mothers with mental illness were excluded. The study was reported by making in line with STROCCS guidelines [22]. In addition, we registered this study on research registry with unique number of 7960, and with hyperlink of https://www.researchregistry.com/browse-the-registry#home/

### 2.3 Study variables

#### 2.3.1 Dependent variable

Post-operative pain.

#### 2.3.2 Independent variables

Socio-demographic factors (Age, Address, Marital status, Educational status, Religion, Monthly income) maternal and operation associated factors (urgency of cesarean section, parity, history of previous cesarean section, ASA status, Duration of operation, Type of anesthesia used, presence of chronic illness).

### 2.4 Sample size determination

The sample size is determined by using a single population proportion formula and considering the following assumptions: proportion of moderate to severe post-operative pain = 85% at the University Gondar. Standard normal distribution value at 95% confidence level of, and margin of error = 5% $=\;\frac{{\left(1.96\right)}^{2}\;x\;0.85\left(1-0.85\right)}{{\left(0.05\right)}^{2}}$

n = 196

Considering a 10% non-response rate, the final total sample size was 216.

### 2.5 Sampling technique

The monthly average cesarean delivery rate for the past 7 months is around 130. Therefore, a total of 1040 cesarean deliveries are expected in the study period, and to get the sample size of 216 by using systematic random sampling, the Sampling interval approximately 5. Then lottery method and the next study participants chose first participant are chosen by passing every fourth patient.

### 2.6 Data collection instruments and questionnaire development

Data were collected by trained junior obstetrics and gynecology residents from mothers who gave birth by c/s, by using interview structured questionnaires and checklists in the labor and maternity wards. Pain outcome variables after surgery were measured by the International Pain Outcome Questionnaire (IPOQ), which was originally developed from the American Pain Society Patient Outcome Questionnaire (APSPOQ) [23]. All questionnaires were prepared in English language, and administered in Amharic language during data collection. For those who had difficulty in understanding of Amharic language, data collectors use translators of local language. Data collection was started after 12 h of surgery and completed at the patient's discharge. Participants' charts were reviewed for records of analgesics administered, type of anesthesia, and duration of surgery.

### 2.7 Data collection procedure

Data collection was conducted through face-to-face interview at maternity wards. A two-day training was given for data collectors and supervisors on pain assessment tools especially the Numeric Rating Scale (NRS) which was used in this study, and how to gather information about pain management. Three data collectors and one supervisor were trained on how to interview depending on the aim of the study, methodology and how to approach to client before the actual data collection is carried out. Before starting the interview, the data collectors read the consent form for the woman. And the interview was continued if she is volunteered to participate.

### 2.8 Quality assurance and management

Data quality assurance was maintained and confirmed during collection, coding, entry and analysis. The questionnaire was adopted from previous studies on pain management practice and prepared in English language. Training and orientation were given for data collectors and supervisors on the data collection procedure. To avoid information, bias those interns, midwifes and the resident who managed the patient under the study was not involved in data collection. Prior to data collection the questionnaire was pretested using 5% of sample size at Adare general hospital to avoid duplications, incomplete information, invalid information and consistency before analysis. After each respondent complete their interview, data collectors checked all questionnaires for completeness before the respondent leave. Data completeness was checked daily by the supervisor and the principal investigator.

### 2.9 Data processing and analysis

After the completion of data collection, the variables were coded and cleaned. The data was entered into the Epi-data software version 3.1 for cleaning for errors and was analyzed by SPSS version 20 (IBM). Descriptive statistic was done and presented with frequency, percentage, mean, and standard deviation. Hosmer and Lemeshow test was used to assess the goodness of fit. Multicollinearity was also checked by the variance inflation factor. Logistic regression was used to determine the association between the independent and dependent variables. A variable with a p-value of less than 0.25 in the bivariate analysis was entered into multivariable analysis, and a p-value less than 0.05 in multivariate analysis was used to declare statistical significance.

### 2.10 Operational definition

*Post-operative pain*: It is pain after the operation until 24 h of the post-operative period. In this study, a patient who has an NRS of 4 and above is classified as having pain [24].

*Numeric rating scale* (*NRS*): A type of pain assessment tool and scale from 0 to 10 (0 = no pain and 10 = worst pain) and instruct the patient to locate their pain status and is commonly used for patients above 8 years. Based on NRS pain is categorized into mild pain (0–3), moderate (4–6) pain, and severe pain (7 and above) [24].

## 3 Results

### 3.1 Socio-demographic characteristics

A total of 216 mothers were enrolled in this study with a response rate of 100%. The mean age of participants were 26.9 (SD ± 4.8) years and ranged from 18 to 38 years. Two hundred eleven (97.7%) of the respondents were married. Out of total participants, 173 (80.1%) were living in urban areas. Regarding educational status of participants, 21 (9.7%) were no formal education, 58 (26.9%) had degree and above educational level. Of the total participants, 86 (39.8%) were housewives, followed by government employees 50 (23.1%) by their occupational status (Table 1).

**Table 1:** Socio-demographic characteristics of parturient who delivered by cesarean section at Hawassa University Comprehensive Specialized Hospital, Southern Ethiopia; 2021 (N = 216).

Variable	Frequency	Percentage (%)
Age		
18-34	193	89.4
≥35	23	10.6
Religion		
Orthodox	75	34.7
Muslim	62	28.7
Protestant	75	34.7
Others	4	1.9
Ethnicity		
Sidama	63	29.2
Oromo	67	31.0
Others^a^	86	39.8
Educational status		
No formal education	21	9.7
Primary	53	24.5
Secondary	84	38.9
Higher	58	26.9
Marital status		
Married	211	97.7
Divorced/widowed	4	2.3
Residence		
Urban	173	80.1
Rural	43	19.9
Occupation		
Housewife	86	39.8
Merchant	21	9.7
Gov't employee	50	23.1
Private worker	42	19.4
Farmer	17	7.9
Monthly income (ETB)		
<5000	72	33.3
5001–10,000	85	39.4
≥10,001	59	27.3

Key:

a Amhara, Gurage, Silte, Tigre.

### 3.2 Maternal characteristics and operation related conditions

The majority of the cesarean sections 166 (76.9%) were performed on women who arrived in an emergency. The mean time for a cesarean section was 51.94(±16.9) minutes, and 50 (23.1%) of the operations took longer than 60 min. In terms of anesthesia, 193 (89.4%) mothers had a cesarean section under general anesthesia. Seventy-five (34.7%) of the study participants had had one or more previous cesarean sections. Ninety-nine (92.1%) of the women had no history of chronic medical illness. 91 (42.1%) of the study participants were primiparous, and the majority of parturient 205 (94.9%) were ASA II (Table 2).

**Table 2:** Maternal health and intra-operative characteristics of parturient who delivered by cesarean section at Hawassa University Comprehensive Specialized Hospital, Southern Ethiopia; 2021 (N = 216).

Variable	Frequency	Percentage (%)
The urgency of the operation		
Emergency c/s	166	76.9
Elective c/s	50	23.1
Duration of procedure		
< 60 min	166	76.9
≥ 60 min	50	23.1
Type of anesthesia used		
General anesthesia	193	89.4
Spinal anesthesia	23	10.6
Presence of previous C/S		
Yes	75	34.7
No	141	65.3
Parity		
Nulliparous	91	42.1
Multiparous	125	57.9
ASA status		
II	205	94.9
III & above	11	5.1
Presence of chronic illness		
No	199	92.1
Yes	17	7.9

ASA = American Society of Anesthesiology status.

### 3.3 Post-operative pain intensity assessment

**Fig. 1:**
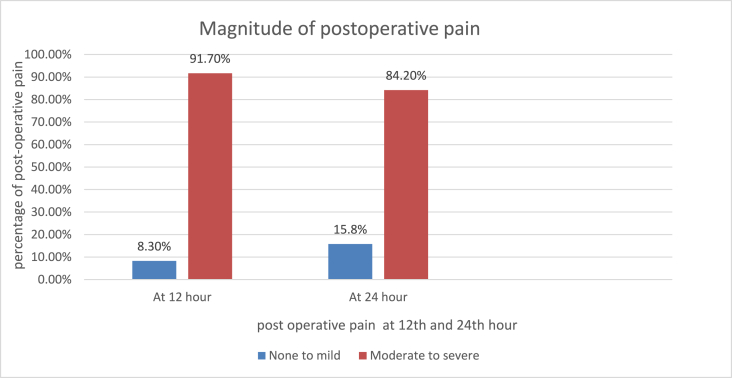
Post-operative pain at 12th, and 24th hours of mothers who gave birth by cesarean section at Hawasa University Comprehensive specialized Hospital, Southern Ethiopia, 2021.

The POP of cesarean-delivery patients was assessed using an NRS on the post-operative period by attending professionals at the postnatal ward at 12, and 24 h after delivery and recorded on their chart. The data collector in this study were record the status of pain based on the patient chart record. Patients with NRS scores of 4 or higher within 24 h of the post-operative period were considered as POP. A large number of patients (91.7%) reported moderate to severe pain at 12 h after surgery, while 84.2% reported moderate to severe pain at 24 h after surgery. In the 24 h post-operative period, the magnitude of post-operative pain was 89.8% (95% CI: 84.7, 93.5) (Fig. 1).

### 3.4 Factors associated with post-operative pain

Parity, history of previous cesarean section, urgency of the operation, duration of the procedure, type of anesthesia, presence of chronic illness, and type of analgesics administered for pain management were candidate variable for multivariable final model. The duration of the procedure, the type of anesthesia used, and the type of analgesics administered were all found to be significantly associated to postoperative pain after cesarean section. The odds of having moderate to severe postoperative pain were 3.62 times higher (AOR: 3.62, 95% CI: 1.33, 15.85) in parturient whose procedure took more than 60 min than parturient whose procedure took less than 60 min. Parturient who underwent cesarean section under general anesthesia were 3.38 times more likely to experience moderate to severe postoperative pain than those who underwent spinal anesthesia (AOR: 3.38, 95% CI: 1.31, 8.7).

Duration of the procedure, type of anesthesia used, and type analgesics administration were found significantly associated with postoperative pain after cesarean section. The odds of having moderate to severe postoperative pain were 3.62 times (AOR: 3.62, 95% CI: 1.33, 15.85) higher among parturient whose procedure took more than 60 min than those parturient who were less than 60 min. Parturient who had undergo cesarean section under spinal anesthesia were 3.4 times more likely to had moderate to severe postoperative pain than those who were operated under general anesthesia (AOR: 2.38, 95% CI: 1.31, 8.7). Parturient who received single agent analgesia were 2.3 times more likely to have moderate to severe post-operative pain after CS than those who received combination form of analgesia (AOR: 2.3, 95% CI: 1.28, 19.21) (Table 3).

**Table 3:** Factors associated with postoperative pain among mother delivered by cesarean section at Hawassa University Comprehensive Specialized Hospital, Southern Ethiopia; 2021 (N = 216).

Variable	Post-operative pain in 24 h		Odds ratio (95% CI)	
	None to mild, N (%)	Moderate to severe, N (%)	Crude	Adjusted
Urgency of the operation				
Emergency c/s	17(10.3%)	149(89.7%)	1.1(0.32–4.05)	0.9(0.23–3.51)
Elective c/s	5(10%)	45(90%)	1	1
Duration of procedure				
< 60 min	12(7.3%)	154(92.7%)	1	1
≥ 60 min	10(20%)	40(80%)	2.47(1.53–13.08)	**3.62(1.33**–**15.85)**^a^
Type of anesthesia used				
General anesthesia	3(1.6%)	190(98.4%)	1.69 (0.40–7.01)	**3.38 (1.31**–**8.71)**^a^
Spinal anesthesia	19(82.6%)	4(17.4%)	1	1
Presence of previous C/S				
Yes	3(4%)	72(96%)	0.56(0.1–1.6)	0.9(0.22–4.5)
No	19(13.5%)	122(86.5%)	1	1
Parity				
Primiparous	8(8.8%)	83(91.2%)	2.18 (1.23–3.87)	0.96 (0.42–2.20)
Multiparous	14(10.3%)	122(89.7%)	1	1
Presence of chronic illness				
No	20(10.1%)	179(89.9%)	1	1
Yes	2(11.8%)	15(88.2%)	0.8(0.11–7.0)	1.4(0.2–13.3)
Medications administered				
Combined analgesics	20(14.3%)	120(85.7%)	1	1
Single analgesics	2(2.7%)	74(97.3%)	1.13(0.01–12.76)	**2.3(1.28**–19.21)*

Key:

a Statistically significant at p-value <0.05.

## 4 Discussion

The aim of this study was to determine the magnitude of postoperative pain within 24 h of a cesarean section. According to the findings, the overall proportion of moderate to severe postoperative pain within 24 h of surgery was 89.8% (95% CI 84.7, 93.5). At the 12th hour, 91.7% of parturient reported moderate to severe pain, and 84.2% reported severe pain at the 24th hour. This finding was consistent with study conducted in Jimma [25], Gondar [14], and Brazil [26]. The differences could be due to the study area and the timing of the pain assessment. However, this finding is higher than that of a study conducted in Palestine [27], Brazil [28], South Africa [29], Sweden [30], Singapore [31] and Uganda [32]. The possible reason could be a lack of attention to pain management and access to strong analgesics to manage post-operative pain.

The type of anesthesia used in this study was statistically associated with post-operative pain. Parturient who underwent cesarean section under general anesthesia were 3.38 times more likely to experience moderate to severe postoperative pain than those who underwent the procedure under regional anesthesia (AOR: 3.38, % CI: 1.31, 8.71). This finding was supported by studies done in Gondar [2], Singapore [33]. Some scholars agree that spinal anesthesia is superior in terms of reducing the intensity of pain, and other study finding also support our study that general anesthesia is a significant risk factor for developing post-operative pain [2].

The duration of the procedure has been associated with post-operative pain. The odds of having moderate to severe postoperative pain were 3.62 times higher (AOR: 3.62, 95% CI: 1.33, 15.85) among parturient whose procedure took more than 60 min than those whose procedure lasted less than an hour. This finding was in congruence with a study done in Singapore [31]. This could be explained by the difficulty of the procedure, which took a long time, the presence of intra-operative complications, which also affect post-operative pain, excessive manipulations of intra-abdominal organs, and the surgeon's experience. Prolonged surgical duration is associated with increased surgical stress on the body and, most likely, increased tissue trauma. The study also found that the likelihood of having moderate to severe post-operative pain after CS was 2.3 times (AOR: 2.3, % CI: 1.28, 19.21) higher in Parturient who received single agent analgesia than those who received a combination form of analgesia. This finding supported by study done in Stanford [21].

### 4.1 Importance of this study

The results from this study expected to give insight to quantify the level of post-operative pain after cesarean section and whether the pain management of post-cesarean patients is adequate or not, so that it will be used as tool to develop appropriate management guidelines to be used by health institutions providing cesarean section care and related pain management. As a reference to study further about pain management practice.

### 4.2 Limitation and strength

The study is cross-sectional, was conducted at a single institution, and had a small sample size, so it may not be generalizable to the general population. In Ethiopia, there is no national pain assessment scale or pain outcome questionnaire. There is a scarcity of literature on this topic to discuss.

## 5 Conclusions and recommendation

In this study, a large proportion of parturient reported moderate to severe post-cesarean pain within 24 h. The duration of the procedure, the type of anesthesia used, and the type of analgesics administered were all found to be significantly associated to postoperative pain after cesarean section. In resource-constrained settings, emphasis should be placed on implementing interventions that reduce pain and aim to provide high-quality postoperative pain management.

## Ethical approval and consent to participants

Ethical clearance was obtained from Hawassa University, College of Medicine and Health Sciences, institutional ethical review board. Following an approval of IRB, Official letter of co-operation was written to concerned bodies by the Department of Obstetrics and Gynecology, Hawassa University. The individual patients had not subjected to any harm as far as the confidentiality is kept. Moreover, no personal identifier was used on data collection form. The data were accessed by a third person except the principal investigator, and was kept confidentially. Written informed consent was taken from the study participants after briefed about the study. Omitting name of the study participants from the questionnaire help to assure confidentiality of the information. During data collection period COVID19 prevention measures were implemented.

## Sources of funding

The author(s) received no financial support for the study, authorship, and/or publication of this article.

## Authors' contribution

All authors contributed significantly to the study's conception, design, data collection, data analysis, and interpretation of the findings. The authors also contributed to the manuscript's writing, reviewed the draft, and ultimately agreed on the journal in which the article should be published. All authors read and approved the final draft of the manuscript and agreed to accept responsibility for all manuscript's contents under any circumstances.

## Registration of research studies

1. Name of the registry: Research registry.

2. Unique Identifying number or registration ID: 7960.

3. Hyperlink to your specific registration (must be publicly accessible and will be checked): support: https://www.researchregistry.com/help-and-support.

## Guarantor

Abbas Ahmed, Dr Ibrahim Hussen.

## Consent

Written informed was obtained from the patient before data collection.

## Data sharing and availability

Data and materials used in this study are available upon reasonable request from the corresponding author.

## Provenance and peer review

Not commissioned, externally peer-reviewed.

## Declaration of competing interest

All authors declare that they do not have any conflicts of interest.

## List of abbreviations

ASA: American Society of Anesthesiology status
HUCSH: Hawassa University comprehensive Specialized Hospital
IASP: International Association of Pain
NRS: Numeric rating scale

WHOWorld health organization
